# Supplementary material for: The interactome of intact mitochondria by cross-linking mass spectrometry provides evidence for coexisting respiratory supercomplexes
Source: Mol Cell Proteomics. 2017 Dec 8;17(2):216–32. doi: 10.1074/mcp.RA117.000470 (PMC5795388; doi:10.1074/mcp.RA117.000470)
Supplement: Supplemental Data [file supp_RA117.000470_133922_0_supp_23978_fzffwf.zip › spectra_annotation/mito_DR_spectra_annotation/499-1-6-1-14-1.pdf]

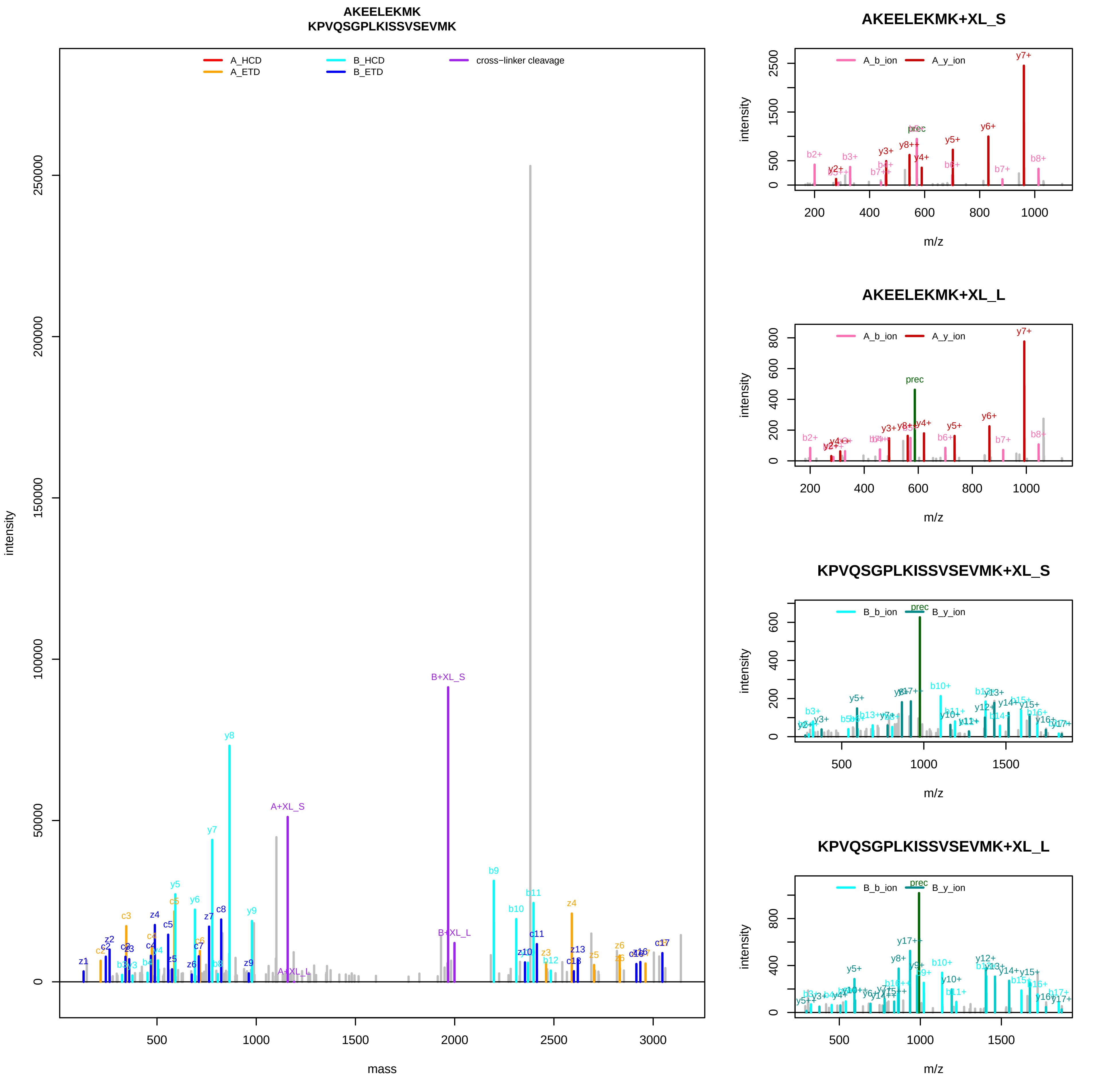

AKEELEKMK
KPVQSGPLKISSVSEVMK
A_HCD
A_ETD
B_HCD
B_ETD
cross−linker cleavage
intensity
mass
0
50000
100000
150000
200000
250000
500
1000
1500
2000
2500
3000
B+XL_S
A+XL_S
B+XL_L
A+XL_L
y8
y7
y5
y6
y9
b9
b10
b11
c11
z4
c3
z4
c5
c8
z7
c7
z2
AKEELEKMK+XL_S
A_b_ion
A_y_ion
y7+
y6+
y5+
y8++
y3+
y4+
prec
b2+
b3+
b6+
b7+
b8+
m/z
AKEELEKMK+XL_L
KPVQSGPLKISSVSEVMK+XL_S
B_b_ion
B_y_ion
b10+
y5+
KPVQSGPLKISSVSEVMK+XL_L
y17++
y8+
y12+
